# Progesterone Receptor Expression in Meningiomas: Pathological and Prognostic Implications

**DOI:** 10.3389/fonc.2021.611218

**Published:** 2021-07-15

**Authors:** Francesco Maiuri, Giuseppe Mariniello, Oreste de Divitiis, Felice Esposito, Elia Guadagno, Giuseppe Teodonno, Marcello Barbato, Marialaura Del Basso De Caro

**Affiliations:** ^1^ Neurosurgical Clinic, Department of Neurosciences and Reproductive and Odontostomatological Sciences, Naples, Italy; ^2^ Section of Pathology, Department of Advanced Biomorphological Sciences, University “Federico II”, Naples, Italy

**Keywords:** meningioma, progesterone receptor, WHO grade, proliferation index Ki 67 MIB1, meningioma recurrence

## Abstract

**Background:**

The progesterone receptor (PR) is variably expressed in most meningiomas and was found to have prognostic significance. However, the correlation with patient age, tumor location, time to recurrence, and pattern of regrowth has scarcely been discussed.

**Methods:**

A surgical series of 300 patients with meningiomas is reviewed. The PR expression was classified as: 0. absent; 1. low (<15%); 2. moderately low (16–50%); 3. moderately high (51–79%); 4. high (≥80%). The PR values were correlated with the patient age and sex, meningioma location, WHO grade, Ki-67 MIB1, recurrence rate, pattern of recurrence (local-peripheral *versus* multicentric diffuse), and time to recurrence.

**Results:**

The PR expression has shown lower rate of high expression in the elderly group (p = 0.032) and no sex difference (including premenopausal *versus* postmenopausal women), higher expression in medial skull base and spinal *versus* other locations (p = 0.0036), inverse correlation with WHO grade and Ki67-MIB1 (p < 0.0001). Meningiomas which recurred showed at initial surgery higher rates of low or moderately low PR expression than the non-recurrent ones (p = 0.0004), whereas the pattern of regrowth was not significant. Higher rates of PR values ≥80% were found in cases with time to recurrence >5 years (p = 0.036).

**Conclusion:**

The higher PR expression in medial skull base meningiomas, the significant correlation with the time to recurrence, the lack of difference of PR expression between premenopausal and postmenopausal women and between local-peripheral *versus* multicentric-diffuse recurrences are the most relevant unreported findings of this study. The rate of PR expression must be included in the routine pathological diagnosis of meningiomas because of its prognostic significance.

## Introduction

The presence of sex steroid hormone receptors in meningiomas is known since about 40 years (1, 2).

Some clinical evidence suggests that sex steroids play a role in the growth of meningiomas; these include the clear female predominance (female/male ratio 2:1), the reported rapid growth during pregnancy (3, 4), and women who receive oral contraceptives or hormone replacement therapy (5, 6). The progesterone receptor (PR) expression is found in variable and often very high rate meningiomas (39 to 88%) in some studies (7–9), whereas the estrogen receptor (ER) expression is lower than 10% and often undetectable. The PR expression was found to be correlated with the WHO grade and recurrence in ours (10) and other studies (11–14), with low expression associated with WHO grade II and recurrence. On the other hand, other factors, including patient age, intracranial tumor location, spinal meningiomas, time to recurrence, and patterns of regrowth, have scarcely been discussed.

This study reviews a surgical series of meningiomas and discusses the pathological correlation and prognostic significance of the PR expression.

## Materials and Methods

### Patient Population

Three hundred fifty-two patients who underwent neurosurgery for intracranial and spinal tumors diagnosed as meningiomas at the neurosurgical clinic of the “Federico II” University of Naples between 2006 and 2016 were reviewed. Two children with neurofibromatosis, five patients with post-irradiation meningiomas, and forty-two patients with recurrences were excluded. Thus, 300 consecutive patients with primary intracranial or spinal meningiomas were included in the study. Besides, the 42 patients with recurrence observed in this period and 33 observed between 2000 and 2006 were included in a recurrence group for a total of 75 patients, all with recurrent intracranial meningiomas.

An ethics committee approval was not required according to local and national legislation.

### Analyzed Factors

The factors analyzed in the study included patient age and sex, meningioma location, WHO grade, PR expression, Ki67 MIB-1, recurrence rate, regrowth or recurrence pattern, and time to recurrence.

According to the patient age, two main groups were identified: group I or elderly ≥70 years old and group II <70 years. For the sex evaluation, the female patients were divided in two groups: A, premenopausal and B, postmenopausal. The tumor location was defined from the review of the magnetic resonance (MR) images and the surgical descriptions. Four groups were identified: group 1 or medial skull base included olfactory groove, ethmoidal-sphenoidal planum, tuberculum sellae, parasellar, clival-petroclival, and foramen magnum meningiomas; group 2 or lateral skull base included middle and lateral sphenoid wing and temporal fossa meningiomas and those of the petrous bone and occipital fossa; group 3 or non-skull base included convexity, parasagittal or falx meningiomas, and those of the tentorium, cerebellar convexity, pineal region and lateral ventricle; group 4 included spinal meningiomas.

The surgical specimens were reviewed independently by two pathologists (MC and EG) who were unaware of the clinical data. The WHO grade was defined according to the 2007 WHO classification (15), which was used at the observation period. The immunohistochemical studies were performed to evaluate the Ki67 MIB-1 and the PR expression. The specimens were fixed in neutral buffered 10% formalin, embedded in paraffin, and cut into sections of 5 mm thickness.

The expression of PR was determined in all specimens with monoclonal antibody against the progesterone (DAKO, Italy 1:400, overnight incubation). The quantitative evaluation was expressed as percentage for positive nuclei among 100 cells, for a total of 500 cells. The percentage of PR positivity was determined by a semiquantitative scoring scale with respect to staining intensity, according to the recommendations for immunohistochemistry of hormonal receptors (16) and slightly modified.

**Figure 1:**
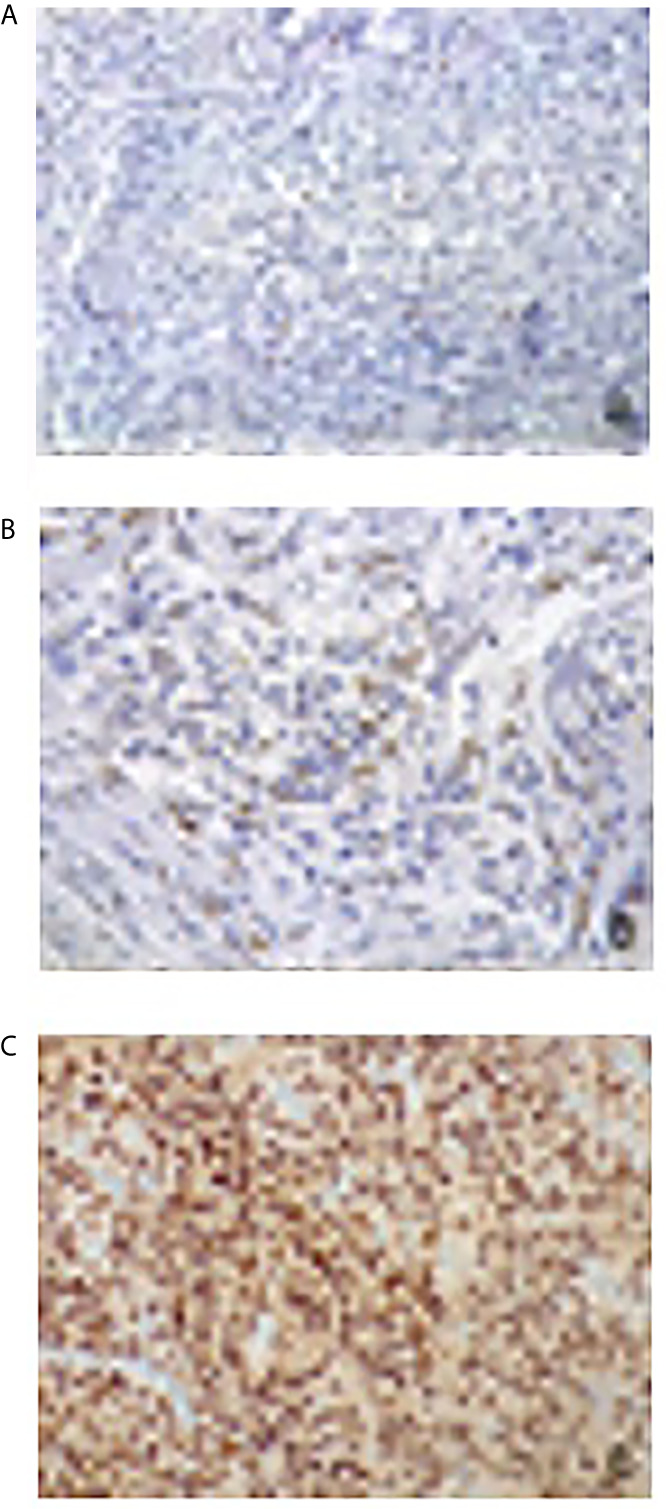
Immunohistochemical evaluation of progesterone receptor antibody expression: nuclear signal respectively in less than 1% **(A)**, in 15% **(B)** and in 95% **(C)** of neoplastic cells (×200 magnification).

The PR expression was graded as follows: 0. absence of positive nuclei; 1. low (<15%); 2. moderately low (16–50%); 3. moderately high (51–79%); 4. high (≥80%) (**Figure 1**).

The expression of Ki67 MIB-1 was evaluated in all specimens by using the monoclonal antibody MIB-1 Immunotech^®^ (DAKO system, dilution 1:1,000, overnight incubation). The streptavidin–biotin system and the diaminobenzidine (DAB) were used for antigen detection and visualization. A specimen of breast carcinoma was used as a positive control. Ki67-LI count was performed by eye counting, taking the average on five adjacent representative fields of neoplastic cells in a hot spot area. The values of Ki67-LI were classified into two groups: group I ≤4%; group II >4%.

The histological types of WHO grade I meningiomas were classified as: meningothelial, transitional, fibroblastic, psammomatous, microcystic, secretory, and chordoid.

The 75 patients with tumor recurrence were classified into two groups: group I (50 cases) with local-peripheral recurrence, in which the recurrence occurred at the previous dural site or at the surrounding dura mater (within 2 cm); group II (25 cases) with multicentric-diffuse recurrence (at variable distance from the initial dural site). The data of these two groups have been compared with those of 100 consecutive patients operated between 2006 and 2010, who did not experience recurrence 9 years or more after the initial surgery.

The analyzed variables included patient age and sex, meningioma location, Simpson grade of surgical resection, PR expression, WHO grade, Ki67 Li.

The patient age was considered as median values; the data stratification between patients ≤70 years and those >70 years was avoided in the analysis of the recurrences because of very different life expectancies and length of the follow-up between the two groups.

In the overall group of 75 patients with recurrence, the PR expression was correlated to the recurrence time (≤5 years *versus >*5 years). Finally, the values of PR expression at the initial surgery were compared to those at the first recurrence.

### Statistical Analysis

The values of PR expression were carefully analyzed and stratified in all cases according to the patient age and sex, tumor location, WHO grade, Ki67-LI, overall recurrence rate, and pattern of recurrence (local and peripheral *versus* multicentric and diffuse). The data were analyzed by one-way ANOVA test or Fisher’s exact test, and p-value was calculated. A p-value ≤0.05 was considered statistically significant. The group of 75 patients with recurrence was studied for PR expression, MIB-1 index, WHO grade, and tumor location, by a multivariate non-parametric statistical tests of hypotheses (Pearson linear correlation test, Spearman R test, Mann–Whitney U test). A Kaplan–Meier test was also performed for the time to recurrence.

## Results

In the overall series of 300 patients with meningioma at first diagnosis, the PR expression was low (0–15%) in 54 (18%), moderately low (16–50%) in 68 (23%), moderately high in 60 (20%) and high (≥80%) in 118 (39%). No cases with complete absence of positive nuclei were found. The data of the PR expression according to the analyzed factors are as follows.

### Patient Age and Sex

The patients were 223 women (74%) and 77 men (26%); their age was <70 years in 225 (75%) and ≥70 years in 75 (25%). The distribution of the PR expression in the age groups (**Table 1**) has shown lower rate of cases with expression ≥80% in the elderly group (p = 0.032). According to the patient sex, no significant difference was evidenced between females and males (**Table 2**). In the female group, 69 (30%) premenopausal and 154 (70%) postmenopausal women were considered separately; the distribution of the PR expression in these last two groups was not significantly different (**Table 2**).

**Table 1:** PR expression and patient age.

PR expression	N. cases	Group 1 (≥70 years) 75 pts	Group 2 (<70 years) 225 pts	Statistical significance group 1 *vs*. group 2
L (0–15%)	54	20 *(27%)*	34 *(15%)*	p = 0.99
ML (16–50%)	68	20 *(27%)*	48 *(22%)*	p = 0.81
MH (51–79%)	60	12 *(16%)*	48 *(21%)*	p = 0.17
H (≥80%)	118	23 *(30%)*	95 *(42%)*	**p = 0.032**

Statistically significant values have been reported in bold.

**Table 2:** PR expression and patient sex.

PR expression	N. cases	Group 1 Females	Group 1A premenopausal women	Group 1B postmenopausal women	Group 2 Males	Statistical significance group 1 *vs*. group 2	Statistical significance group 1A *vs*. group 1B
L (0–15%)	54	41 (18%)	10 (14%)	31 (20%)	13 (17%)	p = 0.55	p = 0.23
ML (16–50%)	68	47 (21%)	16 (23%)	31 (20%)	21 (27%)	p = 0.20	p = 0.63
MH (51–79%)	60	43 (20%)	17 (25%)	26 (17%)	17 (22%)	p = 0.39	p = 0.81
H (≥80%)	118	92 (41%)	26 (38%)	66 (43%)	26 (34%)	p = 0.86	p = 0.24
	300	223	69	154	77		

### PR Expression and Meningioma Location

The meningioma location was at the medial skull base in 72 patients (24%) and at the lateral skull base in 39 (13%); 161 (54%) were non-skull base and 28 (9%) were in the spinal canal. The distribution of the different locations within the four groups is summarized in **Table 3**.

**Table 3:** Meningioma location.

Location	No. of cases
Medial skull base	
- Olfactory groove, planum ethmoidale-sphenoidale	33
- Tuberculum sellae	18
- Parasellar (anterior clinoid and optic canal)	16
- Clivus, petroclival, foramen magnum	5
- Total	72 (24%)
Lateral skull base	
- Middle and lateral sphenoid wings, temporal fossa	14
- Spheno-orbital	16
- Petrous bone, occipital fossa	9
- Total	39 (13%)
Non-skull base	
- Cerebral convexity, parasagittal, falx	137
- Tentorial, cerebellar convexity, pineal	19
- Lateral ventricles	5
- Total	161 (54%)
Spinal	28 (9%)
Total	300

Medial skull base and spinal meningiomas showed significantly higher rate of cases with high PR expression and lower rate of cases with low expression than the lateral skull base and non-skull base meningiomas (p = 0.0036) (**Table 4**).

**Table 4:** PR expression and meningioma location.

PR expression								
Meningioma location	N. cases	L (0–15%)	ML (16–50%)		MH (51–79%)		H (≥80%)	Statistical significance
Medial skull base	72	5 (7%)	13	(18%)	12	(16.5%)	42 (58.5%)	
Lateral skull base	39	6 (15.5%)	9	(23%)	10	(25.5%)	14 (36%)	Lateral skull base and non-skull base
Non-skull base	161	39 (24%)	39	(24%)	31	(19.5%)	52 (32.5%)	*vs*.
Spinal	28	4 (14%)	7	(25%)	7	(25%)	10 (36%)	medial skull base and spinal
Total	300	54 (18%)	68	(23%)	60	(20%)	118 (39%)	**p=0.0036**

Statistically significant values have been reported in bold.

### PR Expression, WHO Grade, Ki67 MIB-1 and Histological Type

Atypical WHO grade II meningiomas have shown significantly lower rate (18%) of cases with high (≥80%) PR expression; on the other hand, benign WHO grade I tumors mainly showed high PR expression (82% of the examined cases). This correlation was statistically significant (p < 0.0001) (**Table 5**).

**Table 5:** PR expression, WHO grade and Ki67/MIB1.

PR expression	N. cases	WHO grade		Statistical significance	Ki 67/MIB1		Statistical significance
		I	II		≤4%	>4%	
L (0–15%)	54	24 *(47%)*	30 *(53%)*	p = 0.30	23 *(42%)*	31 *(58%)*	**p = 0.04**
ML (16–50%)	68	32 *(49%)*	36 *(51%)*	p = 0.40	28 *(41%)*	40 *(59%)*	**p = 0.017**
MH (51–79%)	60	41 *(69%)*	19 *(31%)*	p = 0.99	35 *(58%)*	25 *(42%)*	**p = 0.014**
H (≥80%)	118	97 *(82%)*	21 *(18%)*	**p < 0.0001**	100 *(85%)*	18 *(15%)*	**p < 0.0001**
	300	194 *(65%)*	106 *(35%)*		186 *(62%)*	114 *(38%)*	

Statistically significant values have been reported in bold.

The correlation between PR expression and Ki67 MIB 1 has provided significant differences. Cases with Ki67 LI >4% showed significantly lower rate of high (≥80%) PR expression (p = 0.0001) and higher rates of low (p = 0.04) or moderately low (p = 0.017) expression (**Table 5**). Thus, the study confirms an inverse correlation of the PR expression with both the WHO grade and Ki67 MIB-1.

The most frequent histological type of WHO I meningiomas was transitional (43%) followed by fibroblastic (22%) and meningothelial (15%). Tumors of meningothelial and psammomatous types showed slightly higher rates of high PR expression (76 and 77% respectively) than transitional (63%) and fibroblastic (52%), but with no statistical significance (**Table 6**).

**Table 6:** PR expression and histological type of 194 WHO grade I meningiomas.

PR expression	Meningothelial	Transitional	Fibroblastic	Psammomatous	Microcystic	Secretory	Chordoid
L (0–15%)	3 (10%)	3 (3%)	9 (22%)	2 (9%)	2 (22%)	–	**-**
ML (16–50%)	1 (4%)	14 (17%)	8 (19%)	2 (9%)	2 (22%)	–	2 (50%)
MH (51–79%)	3 (10%)	14 (17%)	3 (7%)	1 (5%)	1 (11%)	–	**-**
H (≥80%)	22 (76%)	52 (63%)	22 (52%)	17 (77%)	4 (45%)	5 (100%)	2 (50%)
Total (194)	29 (15%)	83 (43%)	42 (22%)	22 (11%)	9 (5%)	5 (2%)	4 (2%)
Statistical significance	p = 0.08	p = 0.5	p = 0.09	p = 0.096	p = 0.15		

### PR Expression and Recurrence

The results of the clinical and pathological variables at the initial surgery were compared between the groups of patients with and without recurrence The data are summarized in **Table 7**. The 75 patients with recurrence were 48 (64%) women and 27 (36%) men, with a median age of 55 years at initial diagnosis. The male rate was higher than in the group with no recurrence (25%) but with no significance. No differences of the median values of patient age and sex were evidenced. The analysis of the meningioma location has shown lower rate of medial skull base (13 *versus* 29%) and spinal meningiomas (0 *versus* 8%) and higher rate of lateral skull base meningiomas (31 *versus* 13%) in group I (recurrence). According to the extent of surgical resection, the recurrence groups, as expected, showed significantly lower number of Simpson grade I resection (33 *versus* 61%) and higher rate of grade III resections (27 *versus* 10%) than the no recurrence group. Meningiomas which recurred showed at initial examination higher rate of low and moderately low PR expression (69 *versus* 37%) and significantly lower rate of cases with high PR expression (12 *versus* 43%) (p = 0.0004) than the non-recurrent meningiomas. On the other hand, there were no significant differences between cases with local-peripheral *versus* multicentric-diffuse recurrences (p = 0.5). These data agree with the significantly higher rate of atypical forms (p > 0.00001) and of those with Ki67-LI >4% (p = 0.003) in meningiomas which recurred, as compared to the non-recurrent ones (**Table 7**).

**Table 7:** PR expression, WHO grade, Ki67/MIB1 at initial surgery and recurrence.

Covariates		Group 1 overall recurrence (75 pts)	Group 1A Local-peripheral recurrences (50 pts)	Group 1B Multicentric diffuse recurrences (25 pts)	Group 2 No recurrence (100 pts)	Statistical significance group 1 vs. 2	Statistical significance group 1A vs. 1B
**PR expression**							
L (0–15%)	22 *(29%)*		14 *(28%)*	8 *(32%)*	13 *(13%)*	p = 0.99	p = 0.35
ML (16–50%)	30 *(40%)*		18 *(36%)*	12 *(48%)*	24 *(24%)*	p = 0.98	p = 0.15
MH (51–79%)	14 *(19%)*		12 *(24%)*	2 *(8%)*	20 *(20%)*	p = 0.4	p = 0.95
H (≥80%)	9 *(12%)*		6 *(12%)*	3 *(12%)*	43 *(43%)*	**p = 0.0004**	p = 0.5
**WHO grade**							
I		23 *(30%)*	18 *(36%)*	5 *(20%)*	72 *(100%)*	**p = 0.000001**	p = 0.07
II		52 *(70%)*	32 *(64%)*	20 *(80%)*	28 *(28%)*	**p = 0.00001**	p = 0.07
**KI67/MIB1**							
≤4%		27 *(36%)*	22 *(44%)*	5 *(20%)*	62 *(62%)*	**p = 0.003**	p = 0.07
>4%		48 *(64%)*	28 *(56%)*	20 *(80%)*	38 *(38%)*	**p = 0.003**	p = 0.07

Statistically significant values have been reported in bold.

The multivariate non-parametric statistical tests confirm strong correlation between PR expression ≥80%, low WHO grade, and low expression (≤4%) of Ki 67-Li. The WHO grade is the most efficient variable to predict recurrence. The high PR expression (≥80%) is a single efficient predictive factor (p = 0.017).

**Figure 2:**
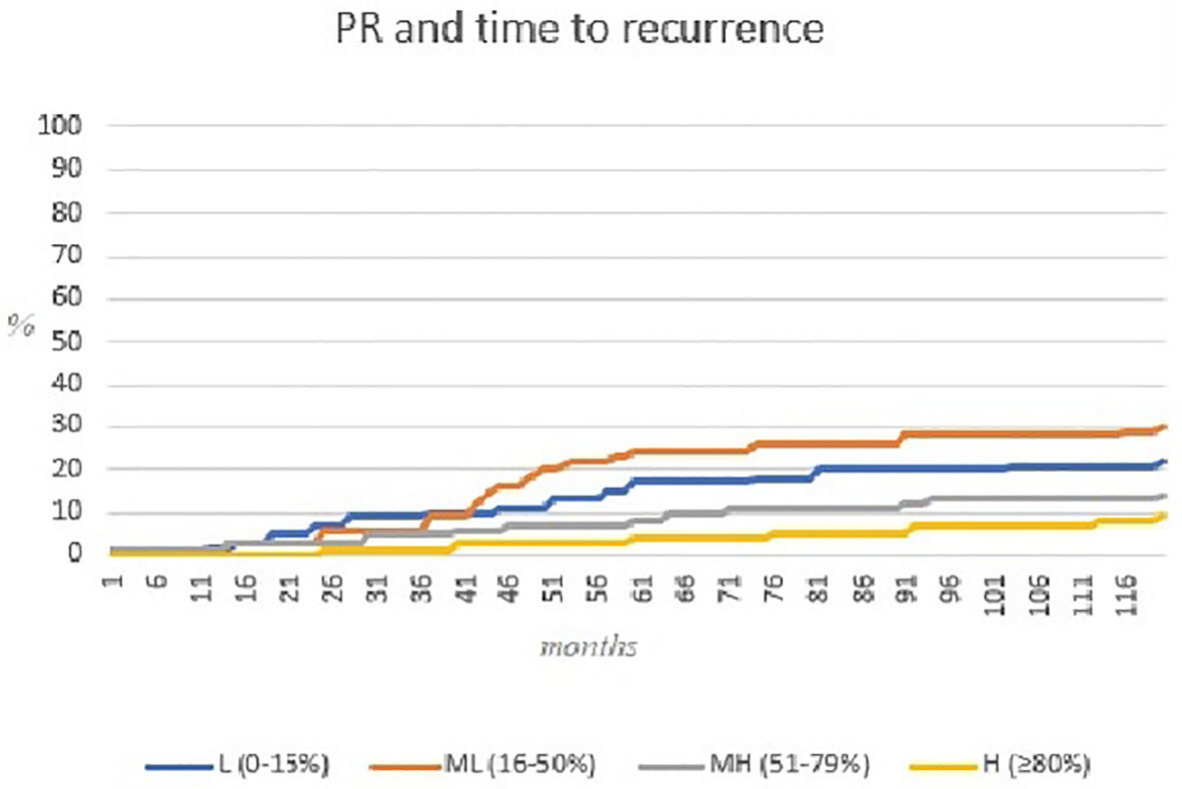
Kaplan–Meier curve representing relation between PR expression and time to recurrence.

The PR expression at initial surgery was significantly correlated with the recurrence time, with higher rate of patients (23 *versus* 8%) with high PR values ≥80% in the group with recurrence time >5 years (p = 0.036) (**Table 8** and **Figure 2**).

**Table 8:** PR expression and time to recurrence (75 pts).

PR expression	Time to recurrence		Statistical significance	Statistical significance groups L+ML vs MH+H
	≤5 years	>5 years		
L (0–15%)	17 (32%)	5 (23%)	p = 0.78	**p = 0.009**
ML (16–50%)	24 (45%)	6 (27%)	p = 0.92
MH (51–79%)	8 (15%)	6 (27%)	p = 0.11	**p = 0.0096**
H (≥80%)	4 (8%)	5 (23%)	**p = 0.036**

Statistically significant values have been reported in bold.

Finally, the PR values of the surgical specimens at recurrence, as compared with those at initial surgery, had the same score (almost unchanged) in 42 cases (56%) and at a lower score in 33 (44%). This finding is associated with rather similar behavior of the Ki67 MIB-1, showing 44 cases (59%) with increased values, from ≤4 to >4%, and 31 (41%) with values in the same subgroup, both at initial surgery and recurrence.

## Discussion

The possible pathological and prognostic implications of the PR expression in meningiomas have been discussed in several studies. However, the role of several factors is still controversial. **Table 9** summarizes the results of 30 studies from the literature that focused on the epidemiological, pathological, and prognostic role of the PR expression in meningiomas (8, 10–14, 17–40).

**Table 9:** Data of 30 reviewed studies on the progesterone receptor expression in meningiomas.

Authors/year	N° of cases	Correlation of PR expression with epidemiological and pathological findings and recurrence							
		Age	Sex	Location	WHO grade	Ki67 MIB1	Mitotic index	Histological type	Recurrence
Magdelenat et al., 1982 (17)	42	n.s.	p = 0.05	n.s.	-	-	-	n.s.	-
Markwalder et al., 1983 (18)	34	n.s.	n.s	n.s.	–	–	n.s	++meningothelial	–
Nagashima et al., 1995 (19)	39	-	p < 0.02	-	p < 0.001	p < 0.05	-	-	-
Hsu et al., 1997 (8)	70	n.s.	–	–	p < 0.001	–	p < 0.0001	n.s	–
Fewings et al., 2000 (11)	62	-	-	-	b.m.	-	-	-	p = 0.013
Perry et al., 2000 (20)	175	–	n.s.	–	p < 0.001	–	–	n.s	–
Das et al., 2002 (21)	90	n.s.	n.s.	-	b.m.	-	-	-	n.s.
Gursan et al., 2002 (22)	110	n.s.	n.s.	n.s.	–	p < 0.05	–	–	–
Strik et al., 2002 (23)	30	-	-	-	b.m.	n.s.	-	-	OR 3.533
Konstantinidou et al., 2003 (24)	51	–	n.s	n.s	p = 0.036	p = 0.041	p = 0.009	++meningothelial p = 0.04	–
Roser et al., 2004 (12)	588	n.s.	n.s	n.s.	p < 0.0001	p < 0.001	-	p < 0.0001	p < 0.0005
Wolfsberger et al., 2004 (25)	82	p=0.05	n.s.	n.s.	n.s.	–	–	++meningothelial p = 0.032	–
Kohronen et al., 2006 (26)	443	n.s.	n.s.	-	n.s.	-	-	-	n.s.
Omulecka et al., 2006 (27)	64	–	–	–	s	–	–	p < 0.05	–
Pravdenkova et al., 2006 (13)	239	-	-	-	p < 0.00009	-	-	-	p = 0.002
Maiuri et al., 2007 (10)	100	–	–	–	–	–	–	–	p < 0.0001
Taghipour et al., 2007 (28)	51	n.s.	p < 0.021	-	s	-	-	-	-
Metellus et al., 2008 (29)	120	–	–	–	–	–	–	–	p = 0.0025
Takey et al., 2008 (30)	57	-	-	-	p = 0.0419	n.s.	-	n.s.	-
Guevara et al., 2010 (31)	42	–	–	–	–	–	–	–	n.s
Kandemir et al., 2010 (32)	53	-	n.s.	-	n.s.	n.s.	-	n.s.	-
Karya et al., 2010 (33)	59	–	–	–	b.m.	–	–	–	n.s.
Shayanfar et al., 2010 (34)	78	-	p < 0.05	-	p < 0.0001	p < 0.0001	-	-	-
Abdelzaher et al., 2011 (14)	60	–	–	–	b.m.	–	–	–	p = 0.028
Tao et al., 2012 (35)	102	-	-	-	-	-	-	-	n.s.
Iplikcioglu et al., 2014 (36)	48	–	–	–	p = 0.01	n.s.	p = 0.002	–	n.s
Mukhopadhyay et al. 2017 (37)	90	-	-	-	p < 0.001	-	-	-	-
Kuroi et al., 2018 (38)	161	–	–	+skull base p=0.00009	–	–	–	–	–
Carvalho et al., 2020 (39)	96	-	-	-	b.m.	n.s.	-	-	n.s.
Portet et al., 2020 (40)	90	–	n.s.	n.s.	n.s.	–	–	n.s.	–
Present study	300	p = 0.032	n.s.	lateral s.b and non-s.b. *VS* medial s.b. and spinal p=0.0036	p < 0.0001	p < 0.0001	-	n.s.	p = 0.0004

n.s., not significant; not studied; s.b., skull base; b.m., only benign WHO grade I meningiomas included; s.g, referred as significant but with no statistical data.

### Definition of the Progesterone Receptor Expression

The score and cut-off values of PR expression have variably been considered in the reviewed reports. Many studies (11, 13, 17, 21, 26, 31, 39) only report negative or positive expression. Others consider as positive only those cases with strong staining in >10% or moderate staining in >50% (12, 34, 37). Two studies (16, 32) used stratification only for cases with positivity in <50% of the cells, whereas cases with >50% positive cells are considered as a unique group. Only three studies (14, 30, 35) have stratified all cases with different positivity, but the employed cut-off values are different. We have used the semiquantitative scoring scale recommended by the Group for Evaluation of Prognostic Factors using Immunohistochemistry, published in 1999 (16); we have only modified the cut-off of the lower expression (15% instead of 10%). We agree that the definition of negative and positive expression is not sufficient. The stratification of the data must be made for all cases with different positivity. In fact, our study shows significant correlation of PR expression with the WHO grade and recurrence only for cases with high PR expression (>80%).

### Progesterone Receptor Expression and Patient Age and Sex

The PR expression of meningiomas in the different age groups is scarcely focused in the literature. Two recent reviews of reported studies on elderly patients do not include data on the PR expression (41, 42). We have found significantly higher rate of PR expression ≥80% in patients aged <70 years (p = 0.032), whereas lower PR values are not correlated. Our results agree with those of Wolfsberger et al. (25); on the other hand, Roser et al. (43) as well as others (8, 17, 18, 21, 22, 26, 28) did not find significant differences between younger and older patients. The discrepancy between our and these studies is likely due to the lesser stratification of the PR values.

The significant correlation between PR expression and patient sex was evidenced in four reviewed studies (17, 19, 28, 34). Others report slightly higher rate of expression in females (14, 22) or in males (25) but with no statistical significance or no relevant sex difference (12, 18, 20–22, 24–26, 32, 40), as in our series. All have considered the overall female group without no relation to the age and the sex female function. We did not find significant differences of PR expression between premenopausal and postmenopausal women. This confirms that the PR expression of meningiomas does not reflect the patient hormonal status.

### Progesterone Receptor Expression and Meningioma Location

Seven reviewed studies (12, 17, 18, 22, 24, 25, 40) did not find significant correlation between PR status and tumor location. However, they consider the overall locations in a unique group. In several studies, as discussed in our recent report (35), the meningioma location was found to be correlated with the WHO grade and Ki67 MIB-1 in several studies which report significantly higher rates of WHO II grades and higher values of Ki67 LI in non-skull base meningiomas.

Only Kuroi et al. (38) reported significantly higher rate of positive PR expression in skull base meningiomas as compared to the non-skull base ones. In our series we have first studied the PR expression of medial skull base and lateral skull base meningiomas as distinct groups; our data show that medial skull base meningiomas have significantly higher rate of cases with higher PR expression and significantly lower rate of cases with low expression than lateral skull base ones.

The higher PR expression of medial skull base and spinal meningiomas, together with the lower values of Ki76-LI (44), may suggest an embryological explanation. Two studies (45, 46) have stated that the meninges around the brain stem develop from the cephalic mesoderm and those of the spinal canal from the somatic mesoderm, whereas the telencephalic meninges develop from the neural crest. This may explain the different PR expression levels and pathological features according to the meningioma location.

These different pathological features have some clinical significance. The skull base meningiomas may have different clinical behavior and recurrence rates. The medial skull base group includes locations, such as olfactory groove, tuberculum sellae, and foramen magnum, with more often slow course and lower recurrence rates (0 to 15%) (47, 48); on the other hand, the recurrence rates are higher for lateral skull base meningiomas (35–40%) (49, 50). This agrees with the different PR expression levels of such locations.

### Progesterone Receptor Expression and Pathological Findings

The correlation between PR expression and pathological findings of meningiomas has largely been discussed, but the reported results are controversial. Among the 30 reviewed studies (**Table 9**), six only included benign WHO grade I meningiomas (11, 14, 21, 23, 33, 39); thus the significance of the WHO grade was not possible. Among the 24 studies including all WHO grades, the correlation between PR expression and WHO grade was studied in 16; 12 found significantly higher rate of cases with high PR expression in benign WHO I tumors and low expression in atypical WHO II ones (8, 12, 13, 19, 20, 24, 27, 28, 30, 34, 36, 37) (**Table 9**). The correlation between PR expression and Ki67 MIB-1 was studied in 10 reviewed series; 5 (12, 19, 22, 24, 34) found significantly lower PR expression in meningiomas with higher Ki67-Li; on the other hand, others (23, 30, 33, 36, 39) did not find significant differences.

The mitotic index was studied in four reports; three of them (8, 24, 36) found significant inverse correlation with the PR expression.

In a recent report (51), we studied the expression of p40, a shorter form of the p53 homolog gene p63, in a series of WHO I and II meningiomas; it was found to be significantly associated with Ki67 LI and recurrence and inversely correlated with the PR expression. All these data confirm that the decrease or loss of the PR expression is associated with histological and biological progression of meningiomas.

The histological subtypes of WHO I meningiomas were studied in 11 reviewed reports; 5 of them (12, 18, 24, 25, 27) have found significantly higher PR expression in the meningothelial ones, with no significant correlations with the other subtypes. In our study the difference of PR expression between the histological subtypes is not significant.

Presurgical information of the PR status of meningiomas, as for other pathological parameters, has recently been obtained with diffusion weighted imaging of magnetic resonance through histogram profiling of apparent diffusion coefficient (ACD) volumes. Skewness and entropy of the ACD are significantly associated with PR expression and Ki 67 LI values (52).

### Progesterone Receptor Expression and Recurrence

Intracranial meningiomas are estimated to recur in 10 to 32% of the cases at 10 years (53–55). Fourteen reviewed studies focused on PR expression and recurrence of meningiomas; seven have found significant inverse correlation, with high recurrence rates in meningiomas with low PR expression at initial surgery (10–14, 23, 29). On the other hand, other studies (21, 26, 31, 33, 35, 36, 39) did not find significant results. Like our previous report (10), the present study confirms the inverse correlation between PR values and recurrence (p = 0.0004); the high PR expression (≥80%) is a single efficient predictive factor (p = 0.017).

The meningioma location may influence the recurrence rate. As discussed in our recent report (44), the medial skull base group includes locations, such as tuberculum sellae and olfactory groove meningiomas, at low recurrence rate; on the other hand, the lateral skull base group includes spheno-orbital meningiomas with dural and bone invasion and higher recurrence rate. This different distribution reflects the different possibilities of achieving resections of Simpson grades I and II.

The present study does not include recurrent spinal meningiomas (only one case in the observation period). Spinal meningiomas very rarely show diffuse growth (56) and are known to recur less frequently than intracranial ones, with reported rates ranging from 0 to 18% (57). Two reports (58, 59) have focused on the PR expression in spinal meningiomas and have found variable positivity in high rate of cases. In a recent study (57) we have first investigated the PR expression in recurrent *versus* non-recurrent tumors, and we did not find significant correlation, with high values in both groups. These data confirm that, differently from intracranial meningiomas, the PR expression is not a predictive factor for spinal meningiomas.

Intracranial meningiomas more often recur at the initial dural site or at the contiguous dural region; however, some patients show multicentric and diffuse recurrences, distant from the initial site, likely from undetected microscopic tumor nodules in distant regions. The reviewed studies which correlate PR expression and recurrence include the overall recurrent tumors, without considering the regrowth pattern. The present study first investigated the PR expression at initial surgery in patients who later experienced local-peripheral *versus* multicentric-diffuse recurrences; we did not find statistically significant differences of PR expression, although the values of Ki67 LI are significantly higher in meningiomas with multicentric and diffuse recurrences. This finding has not previously been reported.

## Conclusion

The higher PR expression in medial skull base meningiomas, the significant correlation with the recurrence time, the lack of difference of PR expression between premenopausal and postmenopausal women and between local-peripheral *versus* multicentric-diffuse recurrences are the main findings of this study.

The immunohistochemical evaluation of the PR expression must be included in the routine histological study of meningiomas, together with the WHO grade and Ki67 LI. Percentages of the expression must be provided, whereas the definition of positive or negative expression is not sufficient.

The well-defined correlation of the PR status with the WHO grade, Ki67 LI, and recurrence is of prognostic significance. For atypical WHO grade II intracranial meningiomas, the low PR expression is a further risk factor of recurrence with the Ki67 LI. For WHO grade I meningiomas, even without high Ki67-LI, the low values of PR expression must suggest a closer follow-up. However, further biomolecular studies will contribute to stratify the group of patients with low PR expression and those at different recurrence risks.

## Data Availability Statement

The raw data supporting the conclusions of this article will be made available by the authors, without undue reservation.

## Ethics Statement

Ethical review and approval was not required for the study on human participants in accordance with the local legislation and institutional requirements. Written informed consent for participation was not required for this study in accordance with the national legislation and the institutional requirements.

## Author Contributions

All authors listed have made a substantial, direct, and intellectual contribution to the work and approved it for publication.

## Conflict of Interest

The authors declare that the research was conducted in the absence of any commercial or financial relationships that could be construed as a potential conflict of interest.
